# A dataset of LC-MS QTOF analysis of potato and mustard crop residue smoke water

**DOI:** 10.1016/j.dib.2018.09.117

**Published:** 2018-10-04

**Authors:** Gaurav S. Dave, Bhemji Galvadiya, Himanshu Bariya, Sudhanshu R. Vyas

**Affiliations:** a Department of Biochemistry, College of Basic Science & Humanities, Sardarkrushinagar Dantiwada Agricultural University, Sardarkrushinagar, 385506, India; b Department of Biotechnology, Hemchandracharya North Gujarat University, Patan, India

**Keywords:** Smoke water, LC-MS QTOF, Crop residue

## Abstract

This data article comprises of the total LC-MS QTOF analysis of smoke water prepared from potato and mustard crop residue. LC-MS QTOF analysis revealed a total of 39 compounds from potato crop residue smoke water, whereas mustard crop residue smoke water exhibited 42 compounds. Molecular formula, mass, RT (retention time) and Area are described in the data presented here. Additionally, different database ID of the identified compounds are mentioned in the data table of potato and mustard crop residue smoke water.

## Specifications table

**Table:** 

Subject Area	Biology
More specific subject area	Agricultural Biochemistry
Type of Data	Table, Image and Graph
How data was acquired	LC-MS QTOF (Agilent Technologies, USA. Model: 6540)
Data format	Raw and analyzed Data
Experimental factors	Sample was filtered through 0.2 µm filter
Experimental features	Smoke water of potato and mustard crop residue was analyzed by standard LC-MS QTOF analysis
Data source location	Sikariya Village (Sardarkrushinagar area) (24.327024 E, 72.295357 N), District Banaskantha, Gujarat, India
Data accessibility	Data is within the article
Related research article	[2]

## Value of the data

• User friendly method of smoke water preparation from potato and mustard crop residue as well as utilized for smoke water preparation from other plant species.
• The data can be used as a reference for analysis and application of crop residue smoke water in various sectors i.e. agriculture (plant hormone, pesticidal, fungicidal, bactericidal molecules), pharmaceutical (anesthetic, anti-allergic, antifungal, antibacterial, anti-oxidative molecules), chemical (free radical source, precursor molecules/main moiety of various chemicals and biochemicals, insecticides, plant hormones), etc.
• The dataset can be utilized for comparative analysis of different verities of potato and mustard crop residue smoke water.

## 1 Data

**Fig. 1:**
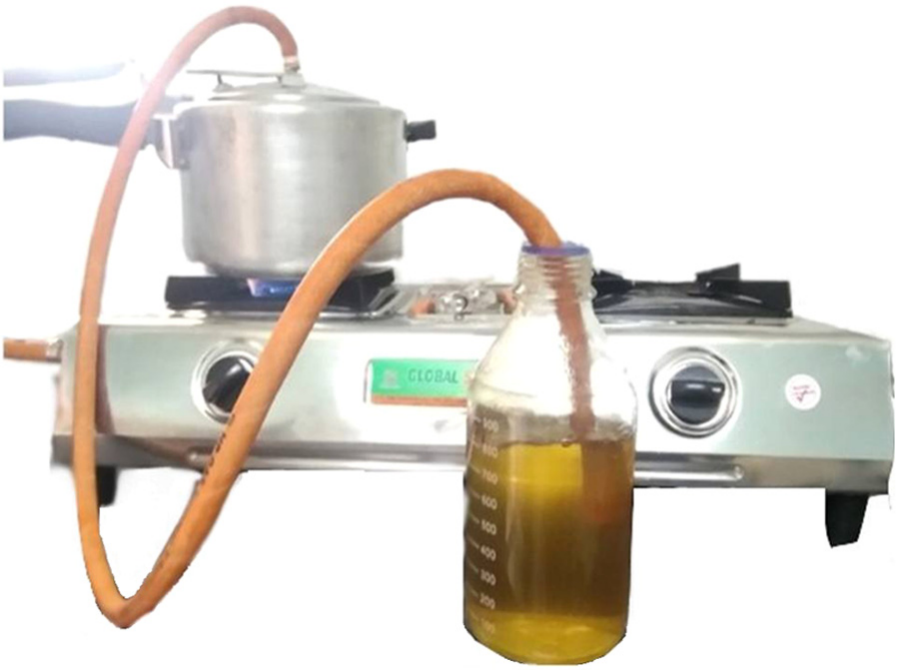
Setup of smoke water preparation at farmer׳s field with house hold facility.

**Fig. 2:**
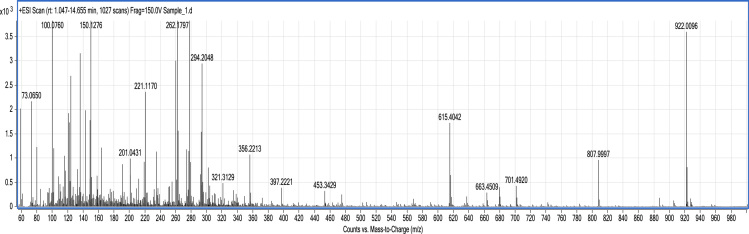
Potato crop residue smoke water MS spectrum.

**Fig. 3:**
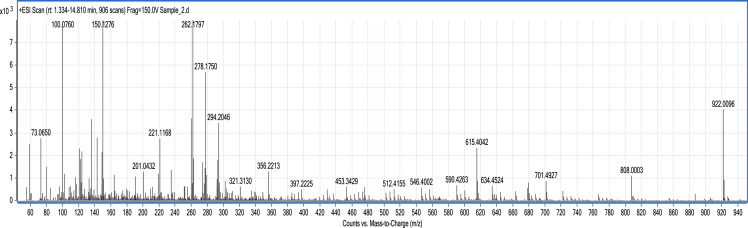
Mustard crop residue smoke water MS spectrum.

The dataset presented is comprised of three figures and two tables. Smoke water preparation method is briefly explained and presented in Fig. 1. Figs. 2 and 3 are MS spectrum of potato and mustard crop residue smoke water, respectively. Tables 1 and 2 are compounds present in potato and mustard crop residue smoke water analyzed and identified by LC-MS QTOF.

**Table 1:** Potato crop residue smoke water LC-MS QTOF analysis.

**Sr. no.**	**Name**	**Formula**	**Mass**	**RT**	**Height**	**Area**	**CAS ID**	**HMP ID**	**KEGG ID**	**Lipid ID**	**METLIN ID**
1	Gallic acid	C_7_H_6_O_5_	170.0207	1.58	5231	20,564	149-91-7				
2	(R)-2,3-Dihydroxypropane-1-sulfonate	C_3_H_8_O_5_S	156.0098	1.608	12,244	74,589			C19675		73285
3	Chromotropic acid	C_10_H_8_O_8_S_2_	319.9659	1.617	3122	16,171	148-25-4		C11323		68990
4	Athidathion	C_8_H_15_N_2_O_4_PS_3_	329.9939	1.644	3345	13,333	19691-80-6		C18964		72723
5	6-Hydroxypseudooxynicotine	C_10_H_14_N_2_O_2_	194.1054	2.313	3207	8747			C01297		63885
6	Pinidine	C_9_H_17_N	139.1356	2.429	15,305	138,985	501-02-0		C10165		68168
7	Desethyletomidate	C_12_H_12_N_2_O_2_	216.0898	2.741	6970	74,435	7036-56-8				2645
8	4-Hydroxy-6-methylpyran-2-one	C_6_H_6_O_3_	126.0316	2.742	8227	108,886	675-10-5		C02752		44653
9	3-Methylbutyraldehyde oxime	C_5_H_11_NO	101.0842	2.781	15,610	117,162	626-90-4		C17255		64529
10	8-Isoquinoline methanamine	C_11_H_11_N	157.089	2.949	36,106	468,035					45036
11	Indole-3-acetaldoxime N-oxide	C_10_H_10_N_2_O_2_	190.074	3.104	14,927	187,160			C17204		71605
12	DMPO	C_6_H_11_NO	113.0838	3.155	19,865	89,651	3317-61-1				64638
13	Phentermine	C_10_H_15_N	149.1203	3.825	18,082	281,934			C07438		43308
14	Muconicdialdehyde	C_6_H_6_O_2_	110.0372	5.133	7509	46,826				LMFA06000011	36533
15	N-(2-Methylpropyl)acetamide	C_6_H_13_NO	115.1	5.271	6726	78,516	1540-94-9	HMDB34203			89774
16	N-(2-Methylpropyl)acetamide	C_6_H_13_NO	115.0996	6.138	16,557	171,447	1540-94-9	HMDB34203			89774
17	Harmalol	C_12_H_12_N_2_O	200.0945	6.412	28,015	503,852	6028-07-5				
18	Mephentermine	C_11_H_17_N	163.1359	7.537	11,523	92,665	1212-72-2		C07889		43865
19	Mephentermine	C_11_H_17_N	163.1355	7.963	21,465	114,876	1212-72-2		C07889		43865
20	Mephentermine	C_11_H_17_N	163.1355	8.065	22,553	156,500	1212-72-2		C07889		43865
21	N-Methylhexanamide	C_7_H_15_NO	129.1155	8.121	8659	28,708			C02722		65819
22	1,2-Benzisothiazol-3(2H)-one	C_7_H_5_NOS	151.0087	9.59	5478	45,464	2634-33-5	HMDB34413			89917
23	3-Nonyl-1H-pyrazole	C_12_H_22_N_2_	194.1779	10.158	8275	84,815	72738-01-3	HMDB34210			89780
24	Uzarigenin3-[xylosyl-(1->2)-rhamnoside]	C_34_H_52_O_12_	652.3436	10.619	5827	35,513	255861-29-1	HMDB36296			91441
25	decanamide	C_10_H_21_NO	171.1619	11.31	2804	19,186				LMFA08010005	46554
26	N-(2,4-Eicosadienoyl)piperidine	C_25_H_45_NO	375.35	11.639	6937	39,539	74267-84-8	HMDB32001			88108
27	(5alpha,8beta,9beta)-5,9-Epoxy-3,6-megastigmadien-8-ol	C_13_H_20_O_2_	208.1461	11.988	4877	44,506	69927-26-0	HMDB34672			90126
28	Phytosphingosine	C_18_H_39_NO_3_	317.2918	12.423	13,272	83,749	554-62-1	HMDB04610	C12144	LMSP01030001	7066
29	Phytosphingosine	C_18_H_39_NO_3_	317.292	12.423	13,272	84,539	554-62-1	HMDB04610	C12144	LMSP01030001	7066
30	2-(3-Phenylpropyl)tetrahydrofuran	C_13_H_18_O	190.1357	12.701	2836	15,009	3208-40-0	HMDB36178			91327
31	Convallasaponin A	C_32_H_52_O_9_	580.3564	13.402	2604	8970	19316-94-0		C08893		67250
32	Sphinganine	C_18_H_39_NO_2_	301.2979	13.719	28,796	239,988	764-22-7	HMDB00269	C00836	LMSP01020001	395
33	Nonoxynol-9	C_33_H_60_O_10_	616.4176	13.884	6362	44,224					43278
34	Sphinganine	C_18_H_39_NO_2_	301.2978	13.958	58,150	639,856	764-22-7	HMDB00269	C00836	LMSP01020001	395
35	N-(3E-hexadecenoyl)-deoxysphing-4-enine-1-sulfonate	C_34_H_65_NO_5_S	599.4586	14.287	4499	49,718				LMSP00000003	53896
36	DG(20:5(5Z,8Z,11Z,14Z,17Z)/0:0/20:5(5Z,8Z,11Z,14Z,17Z)) (d5)	C_43_H_59_D_5_O_5_	665.5075	16.888	2756	18,892				LMGL02010308	4683
37	3Î±,12Î±-Dihydroxy-5Î^2^-chol-8(14)-en-24-oic Acid	C_24_H_38_O_4_	390.2779	16.929	15,185	144,317			C15375		42809
38	N,N-dimethyl-Safingol	C_20_H_43_NO_2_	329.3281	18.45	24,988	565,815				LMSP01080056	53956
39	3-hexanoyl-NBD Cholesterol	C_39_H_58_N_4_O_5_	662.4442	23.455	26,851	275,838					64806

**Table 2:** Mustard crop residue smoke water LC-MS QTOF analysis.

**Sr. no.**	**Name**	**Formula**	**Mass**	**RT**	**Height**	**Area**	**CAS ID**	**HMP ID**	**KEGG ID**	**Lipid ID**	**METLIN ID**
1	Methylthiobenzoic acid	C_8_H_8_O_2_S	168.0244	1.58	11,788	56,665	13205-48-6				2538
2	Gallic acid	C_7_H_6_O_5_	170.0209	1.58	5649	29,649	149-91-7				
3	(R)-2,3-Dihydroxypropane-1-sulfonate	C_3_H_8_O_5_S	156.0104	1.608	14,047	108,311			C19675		73285
4	Bis(2-chloroethyl)ether	C_4_H_8_C_l2_O	141.9947	1.616	162,010	1,088,726	111-44-4		C14688		70262
5	Chromotropic acid	C_10_H_8_O_8_S_2_	319.9659	1.617	4664	28,833	148-25-4		C11323		68990
6	Benzocaine	C_9_H_11_NO_2_	165.0793	1.699	5648	56,717	94-09-7	HMDB04992	C07527		3934
7	Dimethylallyl diphosphate (DMAPP)	C_5_H_12_O_7_P_2_	246.0055	1.722	7387	28,704	1186-30-7		C00235	LMPR01010001	372
8	4-Hydroxy-6-methylpyran-2-one	C_6_H_6_O_3_	126.0324	2.242	4416	17,620	675-10-5		C02752		44653
9	N-Methyl-2-pyrrolidinone	C_5_H_9_NO	99.0683	2.313	23,246	148,444	872-50-4		C11118		68859
10	4-Hydroxy-6-methylpyran-2-one	C_6_H_6_O_3_	126.0318	2.742	12,895	191,624	675-10-5		C02752		44653
11	3-Methylbutyraldehyde oxime	C_5_H_11_NO	101.0842	2.781	12,638	132,453	626-90-4		C17255		64529
12	2-Dodecylbenzenesulfonic acid	C_18_H_30_O_3_S	326.1936	2.854	24,932	304,808		HMDB31031			87362
13	DMPO	C_6_H_11_NO	113.0839	3.155	17,098	281,731	3317-61-1				64638
14	CAY10638	C_16_H_13_NO_3_S_2_	331.0331	3.169	4096	63,483					64838
15	Phentermine	C_10_H_15_N	149.1201	3.825	13,703	175,443			C07438		43308
16	Lys Gln Ile	C_17_H_33_N_5_O_5_	387.2467	4.012	10,454	178,182					16610
17	N-(2-Methylpropyl)acetamide	C_6_H_13_NO	115.0996	5.271	4011	8532	1540-94-9	HMDB34203			89774
18	N-(2-Methylpropyl)acetamide	C_6_H_13_NO	115.1002	6.138	5919	3613	1540-94-9	HMDB34203			89774
19	Azobenzene	C_12_H_10_N_2_	182.0841	7.235	17,093	167,898	103-33-3				
20	ArgArgGln	C_17_H_34_N_10_O_5_	458.2718	7.303	28,835	257,604					16788
21	Mephentermine	C_11_H_17_N	163.1358	7.537	11,022	115,977	1212-72-2		C07889		43865
22	Mephentermine	C_11_H_17_N	163.1351	7.963	8455	40,011	1212-72-2		C07889		43865
23	Mephentermine	C_11_H_17_N	163.1351	8.065	14,785	141,007	1212-72-2		C07889		43865
24	N-Methylhexanamide	C_7_H_15_NO	129.1148	8.121	3910	33,922			C02722		65819
25	8S-hydroxy-2E-Decene-4,6-diynoic acid	C_10_H_10_O_3_	178.0628	9.018	6880	69,087				LMFA01030710	74311
26	2,5-Dimethoxycinnamic acid	C_11_H_12_O_4_	208.0734	9.205	6665	69,742	10538-51-9	HMDB02370			6651
27	1,2-Benzisothiazol-3(2H)-one	C_7_H_5_NOS	151.0086	9.59	4815	36,765	2634-33-5	HMDB34413			89917
28	Monodesmethylpheniramine	C_15_H_18_N_2_	226.1468	10.204	5846	33,503	19428-44-5				1815
29	Uzarigenin 3-[xylosyl-(1->2)-rhamnoside]	C_34_H_52_O_12_	652.343	10.619	7198	44,233	255861-29-1	HMDB36296			91441
30	3׳-N-Acetyl-4׳-O-(10,12-octadecadienoyl)fusarochromanone	C_35_H_52_N_2_O_6_	596.3835	10.628	6889	33,735	136536-84-0	HMDB38566			93237
31	Î±-9(10)-EpODE	C_18_H_30_O_3_	294.2196	11.613	3612	14,741				LMFA02000039	36039
32	Phytosphingosine	C_18_H_39_NO_3_	317.2919	12.423	19,293	121,806	554-62-1	HMDB04610	C12144	LMSP01030001	7066
33	Phytosphingosine	C_18_H_39_NO_3_	317.2922	12.423	19,293	121,806	554-62-1	HMDB04610	C12144	LMSP01030001	7066
34	N-Desmethylselegiline	C_12_H_15_N	173.1198	12.45	2597	13,549	56862-28-3		C15476		2402
35	2-(3-Phenylpropyl)tetrahydrofuran	C_13_H_18_O	190.1358	12.69	7021	80,399	3208-40-0	HMDB36178			91327
36	2-(3-Phenylpropyl)tetrahydrofuran	C_13_H_18_O	190.1358	12.701	7021	80,399	3208-40-0	HMDB36178			91327
37	Curcumenol	C_15_H_22_O_2_	234.1617	13.21	4983	26,060			C16942		71448
38	Nonoxynol-9	C_33_H_60_O_10_	616.4166	13.884	53,287	430,267					43278
39	(25S)-5alpha-cholestan-3beta,6alpha,7beta,8beta,15alpha,16beta,26-heptol	C_27_H_48_O_7_	484.3385	13.949	70,501	558,307				LMST01010327	83928
40	C22 Sulfatide	C_46_H_89_NO_11_S	863.6147	14.113	13,834	83,819				LMSP06020009	41627
41	Polidocanol	C_30_H_62_O_10_	582.4338	14.278	43,576	289,887	3055-99-0		C13493		69582
42	C24 Sulfatide	C_48_H_93_NO_11_S	891.6474	15.211	7981	47,459				LMSP06020013	41630

## 2 Experimental design, materials and methods

### 2.1 Preparation of smoke water

Smoke water was prepared according to the method described previously [1] with modification in apparatus. Total 5 kg of crop residue of Potato (*Solanum tuberosum)* or Mustard (*Brassica nigra*) were collected and packed in air tight heating vessel, i.e. Pressure cooker of 5.0 L in size. Vapor outlet was replaced with rubber tube (1 mt in length with 10 mm diameter) on lid; other end of tube was immersed in 1 L of double distilled water as shown in Fig. 1. Vessel was heated on burner with full flame for 90 min to burn crop residue into smoke and smoke was collected in 1 L of double distilled water, remaining residue after burning weighted 1.45 kg.

### 2.2 LC-MS QTOF analysis

Chromatographic separation was achieved with Chromatographic system (Agilent Technologies, USA. Model: 6540) with column ZORBAX 300SB C-18, 4.6 × 100 mm 3.5 μm at 25 °C as the stationary phase. The method used a gradient at constant flow rate (0.6 mL min^−1^) combining solvent A (0.1% formic acid/water) and solvent B (acetonitrile), programmed as follows: 0 min, linear change from A–B (95:5 v/v) to A–B (5:95 v/v); 12 min, isocratic A–B (5:95 v/v); 20 min, linear change to A–B (95:5 v/v) 22 min and 25 min, linear change to A–B (95:5 v/v). The extract was injected a volume of 10 μL. Compounds of smoke water were identified using LC-MS-Quadrupled time of flight mass spectrometer. The parameters are presented in Table 3.

**Table 3:** QTOF condition Auto Ms/Ms mode.

**Operating parameters**	**Value**
Ion Mode	Positive, ESI ionization mode
Drying Gas Temperature	325 °C
Drying Gas flow	10 L/min
Vaporize/sheath gas Temperature	350 °C
Chamber	4.23 µA
Capillary	0.051 µA
Nebulizer	45 psig
Fixed Collision Energies	10, 20, 30, 40, 50 V
Precursor per cycle Max	5
Precursor Threshold	400 counts
Scan speed	25,000 counts/spectrum
